# A Field Study on Safety Performance of Apron Controllers at a Large-Scale Airport Based on Digital Tower

**DOI:** 10.3390/ijerph19031623

**Published:** 2022-01-31

**Authors:** Jianping Zhang, Xiaoqiang Tian, Jian Pan, Zhenling Chen, Xiang Zou

**Affiliations:** 1 The Second Research Institute of Civil Aviation Administration of China, Chengdu 610041, China; zhangjp@caacsri.com (J.Z.); chenzhenling@caacsri.com (Z.C.); zouxiang@caacsri.com (X.Z.); 2 Baiyun International Airport, Guangzhou 510470, China; panjian@gdairport.com

**Keywords:** digital tower, safety performance, apron controller, situational awareness

## Abstract

The innovative concept of digital tower provides a new solution for reducing the construction and operation costs of airports with adverse natural environments, poor intervisibility conditions, or sparse traffic. However, it leads to changes in the situational awareness of air traffic controllers and to challenges in safety performance. To research the safety performance of apron controllers at a large-scale airport applying a digital tower, a field study was conducted at Baiyun International Airport in Guangzhou, China. In this study, we established a comprehensive index system from the perspective of situational awareness, which provided measurements on the areas of interests, gaze and physiological features, and vigilance of controllers. Three modules were compared: a physical tower module, a digital tower module with a large panoramic screen, and a digital tower module with a small panoramic screen. The differences in the safety performances of apron controllers are discussed in two aspects: adaptability and reliability. The results indicated that the apron controllers at the three modules performed different cognition patterns, but similar cognition effort was paid toward maintaining performance. Furthermore, the significant vigilance decrement of controllers exists between after-duty and before-duty, but with no significant difference among the three modules. In conclusion, apron controllers at a large-scale airport could obtain effective safety performances based on a digital tower that were no less than those from a physical tower.

## 1. Introduction

Digital tower, also referred to as remote tower, is defined as a geographically independent facility from which aerodrome air traffic service (ATS) is provided principally through indirect observation of the aerodrome and its vicinity, by means of a visual surveillance system [1]. The visual surveillance system includes a number of integrated elements, normally consisting of optical sensors, data transmission links, data processing systems and situation displays providing an electronic visual presentation of traffic and any other information necessary to maintain situational awareness at an aerodrome and its vicinity [1]. Generally, the visual surveillance system relies on the optical system, the Pan–Tilt–Zoom (PTZ) and label presentation (fusion information from multiple surface movement detection sensors presented as a label on the visual surveillance screen, e.g., flight number, status, speed, etc.). The concept of digital tower provides a new solution for reducing the construction and operation costs of airports with adverse natural environments, poor intervisibility conditions or sparse traffic [2,3,4]. The International Civil Aviation Organization (ICAO) has listed remote ATS as an important upgrade module in the “2016–2030 Global Air Navigation Plan” [5]. In recent years, research on and applications of digital tower rapidly expanded in Europe due to the efforts of Single European Sky ATM Research Program (SESAR) [6,7]. In the United States (U.S.), Federal Aviation Administration (FAA) has conducted studies on digital tower and remote control since 2006 using the concept of NextGen Tower (NT) [8]. The applications of digital tower also emerged in China recently. Several digital towers in Xinjiang, Guangzhou, and Yunnan were approved for pilot projects in 2021.

Digital towers lead to changes in controller’s work environments, access to air traffic information, and operational procedures that might influence their situational awareness and may result in challenges in controllers’ safety performances [9,10]. Much research has been conducted on safety performance to ensure that controller’s situational awareness obtained from a digital tower is capable [11]. For example, the German Aerospace Centre (DLR) tested some crucial variables when control tasks were completed with a visual surveillance system by various means including high-fidelity simulation [11,12], and eye gaze recording and questionnaires [13]. Furthermore, much research and many tasks have been carried out to assess human performance in multiple modes (a digital tower module remotely providing ATS for two or more aerodromes at a time) at low-density airports. Moehlenbrink et al. investigated different work organizations and their effects on workload in a digital tower using questionnaires [14]. Li et al. studied the controllers’ visual scan patterns over different systems (visual surveillance system, radar data processing, electronic flight strips, and communications network) within multiple modes, which verified the feasibility of one controller providing services for two airports simultaneously [15]. Kearney et al. compared the workload between a digital tower and a physical tower using the human error template (HET) method and NASA-TLX (Task Load Index) questionnaires, which demonstrated a positive effect of enhanced visualization systems [16]. Notably, much work has been carried out by SEASER Joint Undertaking, including safety assessment and human performance assessment for the application of digital towers at middle- and small-size airports [17].

However, most current research focused on low-density airports, while apron control at a large-scale airport has been seldom reported. Apron control determines the taxiways for aircraft entering the apron area until they reach their parking positions and vice versa [18]. At large-scale airports (especially with multiple runways and multiple terminals), normally, the visual range of a physical tower is very limited due to terminals and other obstacles obscuring the view, but a digital tower can provide a new solution for improving the intervisibility of apron areas. Furthermore, obvious differences exist between apron control at a large-scale airport and aerodrome control at a low-density airport. Apron Controllers at a large-scale airport are required to perform more complex tasks including taxi route planning, conflict resolution, etc. Additionally, they need to continuously pay attention, so they experience higher pressures to provide ATS to more aircraft (dozens per hour). Therefore, strengthening special studies on the safety performance of apron controllers based on a digital tower applied at a large-scale airport is of great significance.

Zou et al. conducted research on the safety concerns of a digital tower applied in a large-scale airport using Hierarchical Task Analysis (HTA) method and a human error template (HET) [19]. However, the adaptability of controllers and their reliability when using the visual surveillance system hadn’t been presented. In this paper, we establish an index system on controllers’ situational awareness for data acquisition and conducted a field study on the safety performances of apron controllers based on a digital tower at a large-scale airport in two aspects: adaptability and reliability. The index system reflects the apron controller’s situational awareness at comprehensive levels: perception, comprehension, and response (or projection) [20]. Compared with laboratorial or simulator studies, field studies take advantage of the actual working states and pressures of controllers [21].

## 2. Method and Experiment

To introduce apron control of a large-scale airport, a comparison with aerodrome control at a low-density airport is necessary. Generally, aerodrome controllers at low-density airports, normally with small scales and simple layouts, are aware of their surroundings based on visual observations of out-the-window (OTW) directly and usually have a long duty time. And usually, there is no independent apron control position. At a large-scale airport, an independent unit or position is usually set specifically to provide apron control services due to high traffic flow and complex apron layout, and controllers’ time on duty does not exceed 2 h. Accordingly, except for voice communication system (VCS), many information systems are deployed for independent apron control, such as surface movement guidance and control systems (SMGCS), visual surveillance system, electronic flight strips (EFS), synthetic information display (SID), etc. SMGCS and visual surveillance systems are applied for monitoring responsible areas, especially blind spots, where direct observations of OTW cannot be made. EFS is applied to record the operation information for flights and to execute acceptance or handover of control responsibility between units or positions, and SID is applied to display various auxiliary information (e.g., meteorological condition, flight plan, etc.) [19,22].

When working in daily apron control works at a large-scale airport, controllers continuously observe screens with the information systems to perceive the elements of the environment. Then the controllers quickly interpret the meanings of each element in their mind. Finally, they project the status of the environment and make the right responses (namely give correct instructions to the crews). In this research, all three concepts involved in situational awareness—perception, comprehension and response (or projection)—were concerned.

Furthermore, the safety performance was analyzed in two aspects: adaptability and reliability. Adaptability is defined as an individual’s capacity to make “appropriate cognitive, behavioral, and/or affective adjustments in the face of uncertainty and novelty” [23]. An individual’s visual parameters and physiological features could reflect their adjustments to uncertainty and novelty (adaptability analysis) [24]. Reliability means the probability of an individual’s successfully completing a task [25]. Vigilance decrement can reflect the operator’s competency for a certain task (reliability analysis) [26,27].

Baiyun International Airport (Baiyun Airport) is a super-large airport with two terminals and three runways, where the number of passengers reached 73 million in 2019. Even with the huge influence of the COVID-19 pandemic, it reached 43 million in 2020, ranking first in the world. Baiyun Airport was the first one approved by the Civil Aviation Administration of China (CAAC) to run a digital tower for remote apron control on trial in April 2021. During the trial period, a physical tower module and a digital tower module run simultaneously (one for providing apron control service and the other one for backup, known as the “shadow pattern”). For a better understanding of the safety performance of apron controllers at a digital tower, the control modules at the physical tower and at the digital tower of Baiyun Airport are compared in this research.

### 2.1. Participants

Apron controllers with practicing certificates working at Baiyun Airport were recruited. In total, twenty-one healthy controllers with three class A medical certificates participated after giving their written informed consent. The participants were told that they were free to withdraw from the study at any time. The participants’ ages ranged between 22 and 34 years old (M = 26.85, SD = 2.50). All of their apron control experiences were 1 or 2 years, but their experiences in air traffic control (ATC) ranged between 1 and 10 years (M = 3.95, SD = 1.80) since the apron control unit was recently set up and the participants used to work at other ATC units. Similar experiences for apron control and normal operation during the period of data acquisition indicated that the individual differences among the participants could be ignored in the study. Additionally, only two certificated apron controllers at Baiyun Airport were females. These two female controllers were not on-duty during our study. Therefore, the data acquired in this study were all from male controllers.

Controllers with fatigue are forbidden from performing control duties according to the rules of civil aviation of China (CCAR-93-R5) [28]. Therefore, all participants were requested to rest adequately before a shift and to report when they felt that their alertness improved enough to perform their duties; namely, when they perceived that they had recovered from the fatigue experienced during prior shift work.

### 2.2. Apparatus

#### 2.2.1. Controlling Module

(1) Physical tower module (PTM)

**Figure 1:**
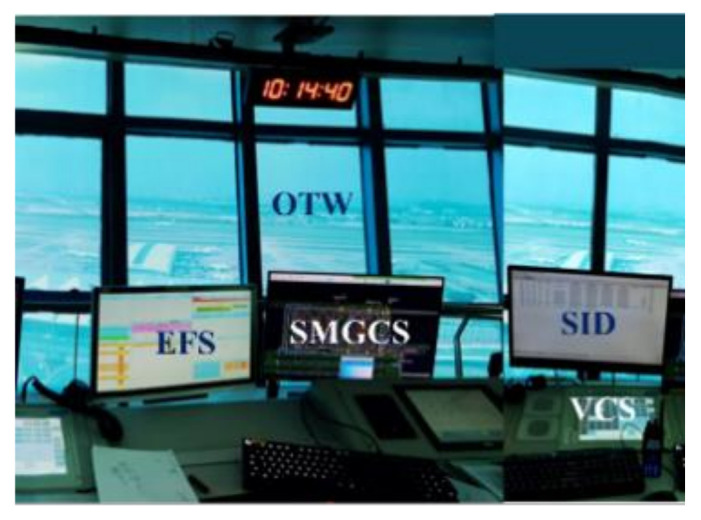
The layout of the physical tower module.

The physical tower module for apron control at Baiyun Airport is mainly equipped with SMGCS, EFS, VCS, and SID. Controllers obtain observations of OTW directly (Figure 1).

(2) Digital tower module (DTM)

**Figure 2:**
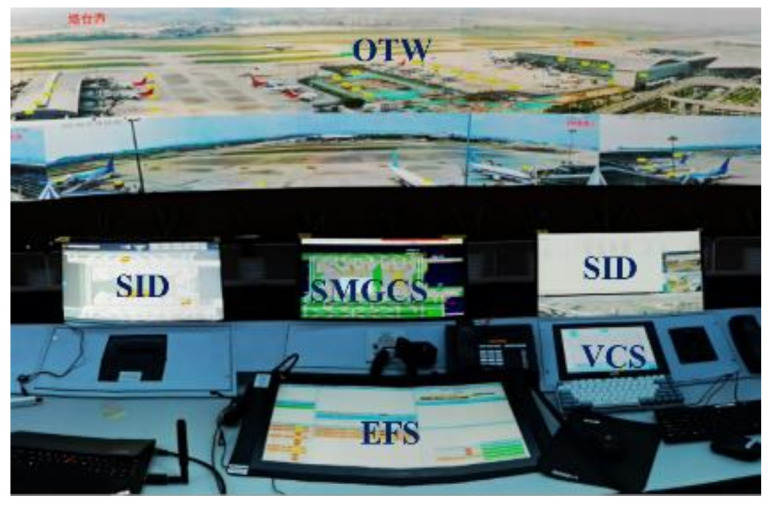
The layout of the digital tower module with a large LED panoramic screen.

A large light emitting diode (LED) panoramic screen with seamless splicing technology is displayed at the digital tower in Baiyun Airport to provide a 360° view of OTW. The LED panoramic screen totals 40.565 m^2^ with a 23.18 m length and a 1.75 m height. The Pan–Tilt–Zoom (PTZ) and label presentation function as part of the display on the panoramic screen. The performance configurations of the whole visual surveillance system are as follows: LED 18,240 × 1350 pixels; LCD 1920 × 1080 pixels; Frames per second (FPS): ≥25 Hz; Time delay between video sensors and the view on panoramic screen: 0.6~0.9 s. Additionally, SMGCS, EFS, VCS, and SID are also equipped at DTM (Figure 2).

**Figure 3:**
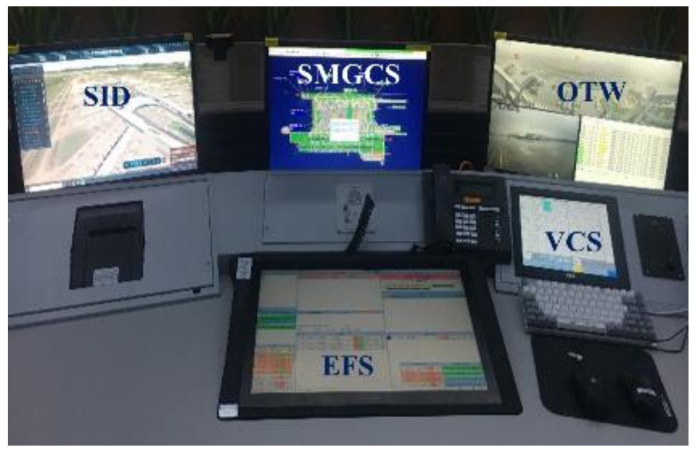
The layout of the digital tower module with a small LCD panoramic screen.

Furthermore, Kearney and Li proposed that a small size of the panoramic video screen may influence controllers to see smaller objects far away from the camera [4]. In order to explore this concern, a new module at the digital tower was added during the trial. In this module, the LED panoramic screen was turned off, and the OTW view was only presented on a 23-inch (1920 × 1080 pixels) LCD screen (Admiral Overseas Corporation (AOC), Wuhan, China) in front of the controllers (Figure 3).

Therefore, the DTM of this study was divided into two categories:

1. A digital tower module with a large LED panoramic screen (LDTM);
2. A digital tower module with a small LCD panoramic screen (SDTM).

#### 2.2.2. Data Acquisition Device

(1) Eye-tracking device

A wearable and light-weight eye-tracking device (Kingfar, Beijing, China) was used to record the participants’ eye movements (the sampling rate was 100 Hz). This device allows participants to freely move their head to perform their tasks. Using the eye-tracking device, fixation count and fixation duration can be recorded to analyze the areas of interests (AOIs) of participants that reflected the controller’s perception of the elements of the environment. Additionally, gaze indexes including blinking, pupil size, saccade, etc. can be recorded to analyze the controller’s cognition effort for comprehension [29,30,31].

(2) Physiological recorder

**Figure 4:**
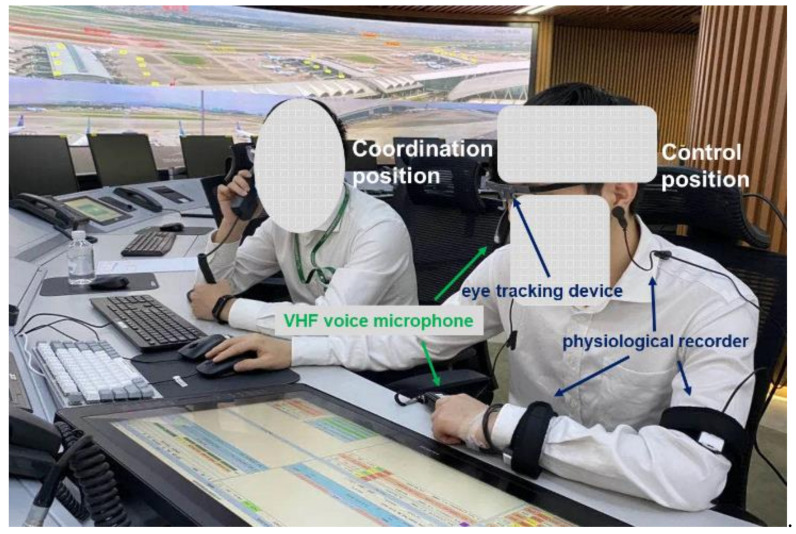
Controllers’ wearing eye-tracking device and physiological recorder for safety performance data acquisition.

A wearable and light-weight human physiological recorder (Kingfar, Beijing, China) was used to collect participants’ physiological data. This device is a group of wearable comprehensive detectors with multi channels of vital signs, including a three-channel ear clip intelligent wearable sensor and a six-channel wrist intelligent wearable sensor (sampling rate was 4096 Hz). It serves as a good solution for monitoring the controller’s physiological parameters during exercise or in a natural state for long durations. The recordings mainly include electrodermal activity (EDA) and heart rate variability (HRV). Recently, physiological measurements have been increasingly used to study controllers’ comprehension of and responses to potential conflicts [24,32,33]. The standard deviation of normal-to-normal R-R intervals (SDNN, a time domain measures of HRV) and the alterations in the conductance of the skin (SC) were recorded in this research. Figure 4 shows the controllers’ wearing the eye-tracking device and physiological recorder for safety performance data acquisition.

(3) Psychomotor vigilance task (PVT)

PVT is a stimulus-response task developed by Dinges and Powell to analyze the vigilance of participants [34]. The standard form of the PVT is a 10-min test containing approximately 90 response trials [35]. However, the 10-min standard duration of the PVT was regarded by many as being too long for applied, operational, or clinical settings [36,37]. For this reason, we developed a modified brief 5-min version of the PVT. When the preset stimulation target appears on the test screen, the participant presses a button, received by the system as an action, and a test record is generated. This measure was proven to have a high sensitivity for vigilance, with many advantages: easy to use, convenient data processing, and low learning effect. Therefore, it has arguably become the most widely used measure of behavioral alertness [38,39]. In this study, reaction time and error rate were measured from PVTs conducted before and after duty to assess the controllers’ ability to respond.

### 2.3. Research Design

#### 2.3.1. Indexes

In this research, the index system on controller’s situational awareness were shown in Table 1.

#### 2.3.2. Statistical Analysis Method

The box plot was employed to screen and remove outliers from the data. The Shapiro-Wilk method was employed to test normal distribution of the data. For analyses of the AOIs, gaze features, and physiological features, a one-way Analysis of Variance (ANOVA) was employed when the variance was homogeneous with Bonferroni correction method for post-hoc comparison, while the Tamhane T2 was employed when the variance wasn’t homogeneous. For the analysis of PVTs, the assumption of sphericity was verified using Mauchly’s test, and repeated measures were employed with Bonferroni correction method for multiple comparison.

#### 2.3.3. Experimental Procedures

**Figure 5:**
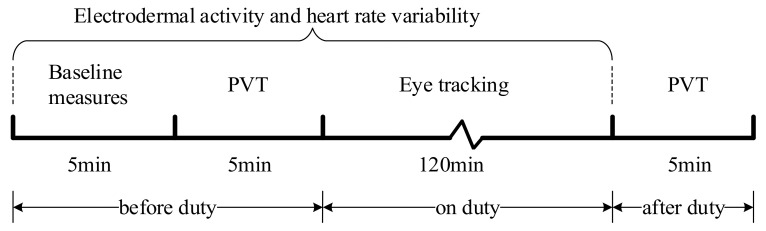
Experimental procedure.

The data collection was conducted at Baiyun Airport in April 2021. All data were collected from the west apron control position. Considering a normal schedule at the apron control center, the daily data collection was arranged at three shifts: 9:30–11:30 in the morning, 13:00–15:00 and 15:00–17:00 in the afternoon, which have a similar traffic volume. Twenty-seven sets of complete and effective records with an experimental procedure timeline shown in Figure 5 were selected from the collected data, nine sets covering the above three different shifts for each of the three modules. In one recorded shift, a controller provided ATS to about 78 aircrafts (M = 77.82, SD = 5.38). No operation error or equipment failure occurred during the recorded time.

The Baiyun Airport apron control center adopts the double duty rule, which means two controllers perform the task simultaneously (one for control and another for coordination) (Figure 4). The one operating the control position is responsible for providing apron control services to aircrafts, and their eye moves across the displays mainly for EFS, SMGCS, OTW, and SID. The one operation the coordination position is responsible for assisting and supervising the person working the control position, and for dealing with coordination with other units or positions. In this research, we only investigated the control position. The timeline of the experimental procedure is shown in Figure 5.

## 3. Results

### 3.1. AOIs

The fixation count and fixation duration were collected with the AOIs (EFS, SMGCS, OTW, and SID) during each recorded shift. The one-way ANOVA or Tamhane T2 was employed to analyze the effect of the modules. Many significant differences were found in the AOI analysis.

(1) Fixation count

The results of the normal distribution tests showed all data on the fixation count corresponded to normal distribution (*p* > 0.05). We compared the influences of the fixation count by the three modules with four AOIs. For the fixation counts on EFS, a significant difference was found F (2, 24) = 9.571, *p* = 0.001. Post-hoc comparison on modules revealed that the participants’ fixation counts on EFS at LDTM are smaller than those at SDTM, *p* = 0.001. For the fixation counts on SMGCS, no significant difference was found. For the fixation counts on OTW, a significant difference was found F (2, 24) = 21.661, *p* < 0.001. Post-hoc comparison on modules revealed that the participants’ fixation counts on OTW at SDTM are smaller than those at LDTM (*p* < 0.001) and PTM (*p* < 0.001). For the fixation counts on SID, a significant difference was found. Post-hoc comparison on modules revealed that the participants’ fixation counts on SID at SDTM are smaller than those at PTM (*p* = 0.032), as shown in Table 2.

(2) Fixation duration

The results of the normal distribution tests showed that all data on the fixation duration corresponded to normal distribution (*p* > 0.05). We compared the influences of the fixation duration by the three modules with four AOIs. For the fixation duration on EFS, a significant difference was found F (2, 24) = 6.503, *p* = 0.006. Post-hoc comparison on module revealed that the participants’ fixation duration on EFS at LDTM is smaller than that at SDTM, *p* = 0.004. For the fixation duration on SMGCS and SID, no significant difference was found. For the fixation duration on OTW, a significant difference was found F (2, 24) = 25.183, *p* < 0.001. Post-hoc comparison on module revealed that the participants’ fixation duration on OTW at SDTM is smaller than at LDTM (*p* < 0.001) and PTM (*p* = 0.001). Additionally, the participants’ fixation duration on OTW at PTM is smaller than at LDTM (*p* = 0.033), as shown in Table 3.

### 3.2. Gaze Features

The pupil diameter, blink rate and saccade rate of each set were collected. The results of normal distribution tests showed that all data on the gaze features corresponded to normal distributions (*p* > 0.05). The one-way ANOVA or Tamhane T2 was employed to identify significant differences, as shown in Table 4.

No significant difference was found in both pupil diameter and blink rate among the three modules. Nevertheless, a significant difference for controller’s saccade count was found F (2, 24) = 3.909, *p* = 0.034. Post-hoc comparison revealed that the saccade rate at LDTM is higher than that at SDTM (*p* < 0.001).

### 3.3. Physiological Features

Physiological features including SC and SDNN were recorded. The results of the normal distribution tests showed that all data of SC and SDNN corresponded to normal distributions (*p* > 0.05) and that the variances were homogeneous. The one-way ANOVA was employed to identify significant differences, as shown in Table 5.

The results demonstrated that no significant difference was found for controller’s SC and SDNN among the three modules.

### 3.4. PVTs

The PVT data were recorded before and after being on duty for every participant. Reaction time and error rate were recorded as shown in Table 6. The results of the normal distribution tests showed that the data of reaction time and error rate corresponded to normal distributions (*p* > 0.05). The results of the multiple comparison analysis with repeated measures are shown in Table 7.

As shown in Table 7, the effect of duty on reaction time (*p* < 0.001) and error rate (*p* < 0.001) were significant which revealed that the vigilance before-duty were less significantly than after-duty (vigilance decrement). However, no significant effect of module on reaction time and error rate was found. And no significant interaction was found between duty effect and module effect.

## 4. Discussion

In this section, we discuss the safety performances of apron controllers based on a digital tower from the perspective of situational awareness. The differences in the safety performances of apron controllers working at the digital tower and those working at physical tower were compared in two aspects: adaptability and reliability.

### 4.1. Adaptability

According to the AOI analysis, apron controllers at the three modules performed different cognition patterns to obtain the air traffic information. Specifically, the fixation counts of EFS at SDTM are higher than those at LDTM, while the fixation counts of OTW at SDTM are smaller than those at LDTM and PTM. And the fixation counts of SID at SDTM are smaller those at PTM. Regarding fixation duration, it is similar to fixation count except for those of OTW at PTM is smaller than at LDTM and no significant difference was found for SID among the three modules. Regarding the controllers’ gaze features and physiological recordings, even though the saccade rate under SDTM was found smaller than that of LDTM, which may be partly due to the difference in the size of panoramic video screen, no other significant differences were found among the three modules. Therefore, it could be inferred that their cognition effort kept the similar trend.

### 4.2. Reliability

Conventional apron control at a physical tower, where controllers provide ATS based on direct visual observation, has been maintained for a long time and is considered feasible and reliable. However, the digital tower changes cognition patterns to the controllers by replacing direct visual observation with various information systems. The results of PVTs showed that 2-h continuous shifts caused a significant vigilance decrement for controllers between after-duty and before-duty, but no significant difference was found among the three modules.

## 5. Conclusions

As a new solution for reducing the construction and operation costs, the research on and applications of digital tower for aerodrome ATS were rapidly increasing around the world. Digital towers lead to changes in the situational awareness of controllers and to challenges in safety performance. To improve field studies on the safety performance of apron controllers based on a digital tower applied at a large-scale airport, we established a comprehensive index system from the perspective of situational awareness and analyzed the differences in the safety performances of apron controllers among the three modules (PTM, LDTM, and SDTM). The field study was conducted at Baiyun Airport using quantitative methods, where the AOIs, gaze and physiological features, and vigilance of controllers were respectively measured. The main findings included the following: (1) From the perspective of adaptability, the apron controllers at three modules performed different cognition patterns, but similar cognition effort was paid toward maintaining performance. (2) From the perspective of reliability, there was a significant vigilance decrement for controllers between after-duty and before-duty, but no significant difference was found among the three modules. The findings indicated that apron controllers at a large-scale airport could obtain effective safety performances based on a digital tower that were no less than those from a physical tower.

It should be noted that this field study was only conducted at Baiyun Airport, and with a limited sample size. Future studies should extend the discoveries at more airports and recruit more subjects. And laboratorial or simulator studies should also be employed with a flexible design of different workload levels or different stages of situational awareness.

## Figures and Tables

**Table 1:** Index system on controller’s situational awareness.

Device	Situational Awareness	Safety Performance
Eye-tracking device	Fixation count (%): Fixation count as a percentage to the total fixations within the AOI	Adaptability
	Fixation duration (%): The percentage of time fixated on the AOI from the total duration	
	Pupil diameter (mm): Average pupil diameter within the segment	
	Blink rate (N/s): Number of blinks per second of each individual within the segment	
	Saccade rate (N/s): Number of saccades per second within the segment	
Physiological recorder	SC (μS): The alterations in the conductance of the skin	
	SDNN (ms): The standard deviation of normal-to-normal R-R intervals	
PVT	Reaction time (ms): The reaction time between stimulus and response in a PVT test	Reliability
	Error rate (%): The percentage of correct responses in a PVT test	

**Table 2:** The results and Comparisons of fixation count.

Index	Module	N	M	SD	One-Way ANOVA		Tamhane T2
					Significance	Post-Hoc	
EFS	LDTM	9	22.69	4.44	F (2, 24) = 9.572, *p* = 0.001	LDTM vs. SDTM **, *p* = 0.001	—
	SDTM	9	32.84	5.53			
	PTM	9	28.37	4.77			
SMGCS	LDTM	9	34.37	6.83	—	—	No significant difference was observed
	SDTM	9	39.32	3.11			
	PTM	9	36.25	2.56			
OTW	LDTM	9	38.82	5.70	F (2, 24) = 21.661, *p* < 0.001	SDTM vs. LDTM **, *p* < 0.001; SDTM vs. PTM **, *p* < 0.001	—
	SDTM	9	24.14	5.03			
	PTM	9	28.19	3.73			
SID	LDTM	9	4.12	1.48	—	—	SDTM vs. PTM *, *p* = 0.032
	SDTM	9	3.69	0.97			
	PTM	9	7.19	3.18			

N: number; M: mean; SD: standard deviation. A *p*-value < 0.05 was considered to indicate statistical significance (*: *p* < 0.05; **: *p* < 0.01). The same to all tables below.

**Table 3:** The results and comparisons of fixation duration.

Index	Module	N	M	SD	One-Way ANOVA		Tamhane T2
					Significance	Post-hoc	
EFS	LDTM	9	24.84	6.03	F (2, 24) = 6.503, *p* = 0.006	LDTM vs. SDTM **, *p* = 0.004	—
	SDTM	9	34.22	6.18			
	PTM	9	28.72	4.19			
SMGCS	LDTM	9	34.70	7.47	—	—	No significant difference was observed
	SDTM	9	38.68	2.19			
	PTM	9	36.08	6.44			
OTW	LDTM	9	36.50	3.45	F (2, 24) = 25.183, *p* < 0.001	SDTM vs. LDTM **, *p* < 0.001; SDTM vs. PTM **, *p* = 0.001; PTM vs. LDTM *, *p* = 0.033	—
	SDTM	9	22.94	4.60			
	PTM	9	28.24	4.13			
SID	LDTM	9	3.96	1.77	—	—	No significant difference was observed
	SDTM	9	3.97	1.04			
	PTM	9	6.97	3.89			

*: *p* < 0.05; **: *p* < 0.01.

**Table 4:** The results and comparisons of gaze features.

Index	Module	N	M	SD	One-Way ANOVA		Tamhane T2
					Significance	Post-hoc	
Pupil diameter (mm)	LDTM	9	3.70	0.26	F (2, 24) = 3.301, *p* = 0.054	—	—
	SDTM	9	3.34	0.34			
	PTM	9	3.69	0.41			
Blink rate (N/s)	LDTM	9	0.53	0.13	—	—	No significant difference was observed
	SDTM	9	0.54	0.25			
	PTM	9	0.50	0.33			
Saccade rate (N/s)	LDTM	9	3.34	0.43	F (2, 24) = 3.909, *p* = 0.034	SDTM vs. LDTM **, *p* < 0.001	—
	SDTM	9	2.45	0.71			
	PTM	9	2.61	0.93			

**: *p* < 0.01.

**Table 5:** The results and comparisons of physiological features.

Index	Module	N	M	SD	One-Way ANOVA	
					Significance	Post-hoc
SC (μS)	LDTM	9	12.65	2.87	F (2, 24) = 0.782, *p* = 0.469	—
	SDTM	9	9.92	6.52		
	PTM	9	11.86	4.18		
SDNN (ms)	LDTM	9	57.34	11.72	F (2, 24) = 2.104, *p* = 0.144	—
	SDTM	9	67.51	11.48		
	PTM	9	55.49	16.40		

**Table 6:** The results of the measurements for PVTs.

Index	Module	N	Before-Duty		After-Duty	
			M	SD	M	SD
Reaction time (ms)	LDTM	9	391.35	58.65	430.80	47.65
	SDTM	9	457.08	61.63	502.04	77.14
	PTM	9	443.62	72.77	479.53	68.21
Error rate (%)	LDTM	9	2.89	2.29	5.24	2.45
	SDTM	9	4.04	3.94	7.56	5.24
	PTM	9	4.46	2.92	6.22	2.72

**Table 7:** The multiple comparison of PVTs.

Index	Multiple Comparison Analysis Using Repeated Measures		
	Duty Effect	Module Effect	Interaction
Reaction time (ms)	F (1, 24) = 59.991, *p* < 0.001 **	F (2, 24) = 2.799, *p* = 0.081	F (2, 24) = 0.259, *p* = 0.774
Error rate (%)	F (1, 24) = 57.515, *p* < 0.001 **	F (2, 24) = 0.669, *p* = 0.522	F (2, 24) = 2.388, *p* = 0.113

**: *p* < 0.01.

## Data Availability

The data presented in this study are available from the corresponding author upon reasonable request.
